# Attachment as an organizer of behavior: implications for substance abuse problems and willingness to seek treatment

**DOI:** 10.1186/1747-597X-1-32

**Published:** 2006-11-02

**Authors:** Kristin M Caspers, Rebecca Yucuis, Beth Troutman, Ruth Spinks

**Affiliations:** 1 Roy J and Lucille Carver College of Medicine, Department of Psychiatry, University of Iowa, Iowa City, IA, 52242, USA

## Abstract

**Background:**

Attachment theory allows specific predictions about the role of attachment representations in organizing behavior. Insecure attachment is hypothesized to predict maladaptive emotional regulation whereas secure attachment is hypothesized to predict adaptive emotional regulation. In this paper, we test specific hypotheses about the role of attachment representations in substance abuse/dependence and treatment participation. Based on theory, we expect divergence between levels of maladaptive functioning and adaptive methods of regulating negative emotions.

**Methods:**

Participants for this study consist of a sample of adoptees participating in an ongoing longitudinal adoption study (n = 208). The Semi-Structured Assessment of the Genetics of Alcohol-II [41] was used to determine lifetime substance abuse/dependence and treatment participation. Attachment representations were derived by the Adult Attachment Interview [AAI; [16]]. We constructed a prior contrasts reflecting theoretical predictions for the association between attachment representations, substance abuse/dependence and treatment participation.

**Results:**

Logistic regression was used to test our hypotheses. As predicted, individuals classified as dismissing, preoccupied or earned-secure reported the highest rates of substance abuse/dependence. Individuals classified as dismissing reported significantly lower rates of treatment participation despite their high rates of substance abuse/dependence. As expected, the continuous-secure group reported lowest rates of both substance abuse/dependence and treatment participation.

**Conclusion:**

The findings from this study identify attachment representations as an influential factor in understanding the divergence between problematic substance use and treatment utilization. The findings further imply that treatment may need to take attachment representations into account to promote successful recovery.

## Background

Attachment theory provides clinicians and researchers alike a method of examining the impact of early experiences on later adjustment [1-6]. According to attachment theory, early experiences with caregivers are transformed into internal mental representations of attachment during childhood and adolescence [3,7-10]. Internal models of attachment are theorized to consist of beliefs about the self and others from which rules are derived and used to guide behavior [4-7,10,11]. Attachment representations are further believed to affect behavior by influencing the intensity of emotional experience and subsequent attempts at emotional regulation [3,8]. In this paper, we utilize the concept of attachment as an organizational construct from which predictions about substance use problems and willingness to seek treatment can be derived [12].

Attachment theory allows for specific predictions about quality of early experiences with caretakers and the effect of these experiences on future behavior and relationships [3,13]. Internal working models of attachment are constructed from repeated interactions with caretakers and are derived from the responsiveness of caretakers during episodes of distress [4,5,7,14,15]. These internal representations are hypothesized to act as filters for future relationships and experiences [1,7,9,13,16]. For example, responsive and supportive behaviors from caretakers are thought to produce secure attachment representations, which are hypothesized to result in openness to emotional experiences and a willingness to engage in creative and productive emotional regulation. In contrast, unsupportive caretaking (e.g., rejection, neglect) during childhood is thought to be characteristic of dismissing attachment. As a result, individuals classified as dismissing are most commonly characterized as engaging in emotional distancing and greater reliance on the self rather than others. Finally, inconsistent support from caretakers during childhood is most often associated with preoccupied attachment which is thought to produce persistent anxiety towards interpersonal relationships and exaggerated levels of negative affect.

Attachment representations show predictive associations with a wide-range of pathological behavior including personality disorder(s), mood disturbance and psychopathy [3,8,13,17-24]. Empirical support of the association between attachment and problematic substance use is less explored and most studies assess self-reported attachment styles [25-30] rather than internal mental representations of attachment as derived from instruments such as the Adult Attachment Interview [16]. Studies in which attachment representations (e.g., internal working models of attachment) are assessed typically rely on clinical samples, suffer from limited sample sizes, or provide inconsistent results [21,24,3,32]. For example, Riggs and Jacobvitz [32] failed to demonstrate a significant association between substance abuse problems and organized attachment, whereas unresolved attachment surrounding abuse was predictive. In contrast, Rosenstein and Horowitz [21] found a trend toward higher rates of substance abuse among adolescents classified as dismissing when compared to adolescents classified as preoccupied. Consistent with Rosenstein and Horowitz [21], Allen, Hauser, and Borman-Spurrell [31] found a significant positive association between problematic substance use and scales most often attributed to a dismissing state of mind (e.g., derogation of caretakers) and a negative association with scales most often associated with preoccupied attachment (e.g., involving anger). However, Allen et al. [31] failed to find a significant overall effect of attachment category on substance abuse.

Preliminary analyses of data presented in this paper show significant associations between attachment representations and reports of illicit substance use within a non-clinical sample [33]. We found significantly higher rates of illicit substance use among individuals classified as dismissing or preoccupied when compared to individuals classified as secure. Although our preliminary analyses provide support for a potential role of attachment in substance use, there were several limitations to our study. First, analyses were limited to the prediction of ever using a substance which combines experimental and problematic users into a single indistinguishable group (i.e., users). Second, variation in inferred childhood experiences within the secure group was ignored (i.e., earned- versus continuous-secure). Although the validity of such a distinction has been questioned, we believe separation of the secure group into continuous versus earned-security is warranted due to differences in rates of psychopathology between the two groups. Earned-security is associated with higher rates of mood disturbance which might increase risk for problematic substance use [34-37]. Finally, the illicit substance use group combined marijuana and non-marijuana substances while alcohol was excluded from the analyses. Therefore, one primary goal of this paper is to examine the association between attachment representations and problematic substance use within a large, non-clinical sample.

A second goal of this project is to examine the association between attachment representations and treatment participation. The presence of substance abuse problems increases the likelihood that professional intervention will be required. In addition to predictions about openness to and intensity of emotional experience, attachment theory can be used to make hypotheses about orientations towards interpersonal relationships and, ultimately, willingness to seek professional intervention [3]. For example, the experience of supportive and sensitive parenting characteristic of individuals classified as secure is thought to promote a view that others' are available during episodes of distress which will lead to a greater likelihood of seeking support. Experiences of rejection or neglect typically associated with dismissing attachment, on the other hand, is hypothesized to promote feelings of self-reliance and a view that others' are unavailable when distressed. Consequently, individuals classified as dismissing may be less likely to turn to others for assistance. Finally, inconsistent parenting, which is characteristic of preoccupied attachment representations, is thought to produce a hypervigilance towards others' coupled with a continued dissatisfaction with support received. Therefore, individuals classified as preoccupied are expected to report higher rates of seeking professional support due to the unsuccessful impact of intervention.

Although researchers have studied the interplay between attachment representations and therapeutic quality, very little research has been conducted on the impact of attachment on the willingness to seek treatment [38-40]. We are aware of only a single study that examined attachment representations and likelihood to seek treatment. Riggs, Jacobvitz, and Hazen [11] report significant associations between lifetime history of psychotherapy and attachment representations. Individuals classified as dismissing reported the lowest rates of psychotherapy whereas individuals classified as secure reported the highest. Riggs et al. did not distinguish between earned- and continuous-secure which may contribute to high rates of therapy within the secure group [11]. Concurrent examination of the association between attachment representations, substance use problems, and willingness to seek treatment will further advance substance abuse interventions.

In summary, attachment theory allows for specific predictions about adaptive and maladaptive behavior in adulthood. We test two primary hypotheses in this paper. Our first hypothesis predicts that individuals classified as dismissing, preoccupied or earned-secure will report higher rates of problematic substance use than individuals classified as continuous-secure. This proposition arises from the role of both inferred childhood experiences and attachment representations in maladaptive emotional regulation [3,13]. We predict high rates of lifetime substance abuse/dependence among individuals classified as earned-secure, despite their secure state of mind, due to the influence of inferred negative childhood experiences on substance use. Although ratings on childhood experiences derived from the Adult Attachment Interview [16] are not veridical with actual experience, we hypothesize that the negative mood proposed to account for the view on childhood experiences will increase the likelihood of problematic substance use [34,36]. Problematic substance use among individuals classified as dismissing or preoccupied, on the other hand, is hypothesized to result from an insecure working model of attachment which is thought to promote maladaptive approaches to emotional regulation (e.g., substance abuse). Our second hypothesis predicts different rates of treatment participation as a function of attachment representations. We anticipate low rates of treatment participation by individuals classified as dismissing, despite predicted high rates of substance use problems, due to persistent devaluing of relationships common among this group. We predict high rates of treatment participation among individuals classified as preoccupied, due to hypervigilance towards distress, and earned-security, due to strong valuing of relationships [1,32].

## Method

### Participants

Participants for this study were recruited as part of an ongoing longitudinal adoption study. Roughly half the adoptees were originally selected due to the presence of psychopathology (e.g., alcohol problems and/or antisocial behaviors) in a birth parent. During the most recent re-interview, participant psychiatric histories were updated and the Adult Attachment Interview [AAI; [16]] was administered (n = 208). Approximately 53% of the sample was female and ranged between 24 and 66 years of age (M = 39, S.D. = 7.95). Average household income was $40,000 to $49,999 per year. The sample was predominantly Caucasian (92%), with the remaining sample comprised of 4% Hispanic, 2% African American, and 1% "Other". Adoptees were adopted by non-relatives within 2 months of age (SD = 5.44) with 67.8% adopted prior to one month and 94.2% adopted prior to 6 months of age.

### Measures

#### Adult attachment classification

The AAI [16] is a semi-structured interview that assesses an individual's attachment representations. Individuals are asked to provide five adjectives describing their childhood relationship with their adoptive mother and father, separately. Participants are also asked to provide experiential support for the chosen descriptors. Questions about parental responses during episodes of emotional upset, illness, and injury are also probed, as are experiences with death and trauma. Finally, the individual is asked to describe changes in and current feelings about their relationship with their parents. Interviews were transcribed verbatim and coded by coders deemed reliable by the lab of Mary Main and Eric Hesse (Rebecca Yucuis and Kristin Caspers, Trained by Deborah Jacobvitz, Austin TX, 2001; Beth Troutman and Jeanne Frederickson, Trained by June Sroufe, Minneapolis, MN, 2002 and 1999, respectively). Approximately half of the AAIs were double-coded. Disagreements were resolved through conference. Overall inter-rater agreement was 94% for the secure versus insecure distinction (κ = .86, p < .001), 91% percent agreement for the organized classifications (κ = .84, p < .001), and 93% agreement for the unresolved/not unresolved classifications (κ = .71, p < .001). Cronbach alphas were equally high for the individual scales ranging from .84 to .93.

The first step in coding attachment representations involves rating inferred childhood experiences with parents. Five parental behaviors are rated on a 9-point scale: loving, rejection, pressure-to-achieve, involving-reversing, and neglect. Loving behavior reflects emotional support and availability. Rejection reflects active rejection or an avoidance of a child's attachment behaviors. Involving-reversing represents role-reversal between parent and child. Pressure-to-achieve reflects parental emphasis on achievement as a key component to the parent-child relationship. Finally, neglect represents parental unresponsiveness to attachment-related behaviors. The method by which ratings for childhood experiences are derived results in estimates of probable experiences with caretakers during childhood and adolescence. The presence of behaviors may be determined by either direct evidence (e.g., provision of comfort during episodes of distress) or the absence of evidence (e.g., no mention of comfort during episodes of distress). Therefore, ratings of childhood experiences are considered inferred rather than reflective of actual behaviors.

The next step in coding is determining attachment representations [16]. The transcript is evaluated for coherency and key indicators for each classification are rated. Three primary organized states of mind are derived from the transcripts: dismissing (Ds), secure (F), and preoccupied (E). Dismissing attachment is characterized by an inability to recall specific memories for positive adjectives used to describe either the mother and/or father. Individuals classified as dismissing often show a high degree of self-reliance, place minimal value on attachment relationships, and portray their childhoods as positive but are unable to provide experiential support. Inferred parental behaviors of rejection and/or neglect are most often associated with dismissing representations. Individuals classified as preoccupied are unable to focus on questions at hand and respond in either a vague or actively angry manner when discussing past or current interactions with their caretakers. These individuals appear as if they are unable to move beyond their childhood experiences, remaining entangled with their parents. Inferred childhood experiences associated with preoccupied attachment most often involve inconsistent behavior and/or involving-reversing. Finally, individuals classified as secure are able to provide experiential support for adjectives provided, whether positive or negative. They are consistent in their portrayal of early experiences, are willing to evaluate past and current relationships, and show valuing of attachment and forgiveness for negative experiences. The secure classification can be further divided into two sub-classifications based on ratings for inferred parental behavior: earned-secure and continuous-secure [32-37]. The earned-secure group is comprised of individuals with low ratings on positive indicators of inferred childhood experiences but demonstrate a secure state of mind. The continuous-secure group is comprised of individuals who experienced a supportive relationship *with at least one *parent and expectedly developed a secure state of mind.

Finally, a category of unresolved/disoriented (U) is assigned when significant lapses in discourse are present during discussions of loss or trauma. A few examples of speech patterns indicative of the unresolved category are confusions of the dead person as living, excessive detail surrounding the event of death or trauma, identifying the self as causal in the death of a loved one or deserving of abuse, or extreme reactions to experiences of loss or trauma. Given a classification of unresolved, subjects are also assigned a corresponding organized classification (*e.g*., U/Ds).

Substance use problems

The Semi-Structured Assessment for the Genetics of Alcoholism – II [SSAGA-II; [41]] was used to collect detailed information about lifetime substance use including alcohol, tobacco, marijuana, and all non-marijuana substances (e.g., cocaine, stimulants, hallucinogens, etc). Lifetime diagnoses of abuse or dependence of alcohol, marijuana, and any illicit drugs were derived from DSM-IV criteria. Overall prevalence rates for each diagnosis were as follows: alcohol dependence (18/208, 9%), alcohol abuse (90/208, 43%), marijuana abuse or dependence (42/208, 20%), and any illicit drug abuse or dependence (35/208, 17%). Fifty-six percent (115/208) reported one or more substance-related diagnosis. Substance abuse or dependence of alcohol, marijuana, or non-marijuana drugs served as independent outcome variables.

#### Mental health treatment

Solicitation of mental health care was determined from the SSAGA-II [41]. Two questions were used, each based on a yes/no response: 1) Have you ever spoken to a professional (e.g., psychiatrist, psychologist, medical doctor, nurse) about any emotional problems and 2) Have you ever received outpatient treatment which includes speaking to a psychiatrist, psychologist, or therapist. Fifty percent (104/208) reported seeing a mental health professional and 38% (79/208) reported receiving outpatient treatment. The majority of individuals sought treatment for emotional problems not related to substance abuse/dependence. Sixty-six percent of those who spoke to a professional about an emotional problem also reported receiving outpatient treatment.

#### Analyses

Logistic regression (SPSS, v. 14.0) was used to examine the association between substance abuse/dependence, treatment participation and attachment representations. We relied on attachment theory to construct orthogonal contrasts. For the prediction of substance abuse/dependence, we predicted higher rates of problematic use among individuals classified as dismissing, preoccupied, or earned-secure when compared to individuals classified as continuous-secure. We also predicted non-significant differences among the former three groups (i.e., dismissing, preoccupied, and earned-secure). Thus, three contrasts were constructed reflecting the following comparisons (assigned values indicated in parentheses): 1) continuous-secure (-1) versus all other classifications (+.333), 2) preoccupied or earned-secure (-.50) versus dismissing (+1) and 3) earned-secure (-1) versus preoccupied (+1). The three contrasts were entered simultaneously into logistic regressions predicting alcohol, marijuana, or non-marijuana substance abuse/dependence.

For the prediction of treatment participation, we hypothesized higher rates of treatment among individuals classified as preoccupied or earned-secure when compared to individuals classified as dismissing or continuous-secure. We also predicted no differences between the former groups (e.g., dismissing and continuous-secure) as well as no differences between the latter groups (e.g., preoccupied and earned-secure). Three contrasts tested our hypotheses in the prediction of treatment participation (assigned values indicated in parentheses): 1) dismissing or continuous-secure (-.50) versus preoccupied or earned secure (+.50), 2) dismissing (-1) versus continuous-secure (+1), and 3) earned-secure (-1) versus preoccupied (+1). The three contrasts were simultaneously entered into a logistic regression predicting lifetime history of treatment participation.

We also tested for the effect of gender, current age, current mood (e.g., depression/anxiety symptoms) and personality disorder on the association between attachment representations, substance use problems, and treatment participation. The parameter estimates for attachment representations were not substantially reduced and remained significant. Therefore, the unadjusted findings are presented.

## Results

### Preliminary analyses

The distribution of the AAI organized classifications differed significantly from expected, χ^2 ^(2) = 31.965, p < .001, with a larger proportion of individuals in the dismissing category (Observed = 39% versus Expected = 24%) and a smaller proportion of individuals in the preoccupied classification (Observed = 8% versus Expected = 18%) [42]. The distribution of the unresolved classification (20%; 39/194), however, was consistent with expectations, χ^2 ^(1) = .558, p = .455.

We examined the validity of our definition of earned-secure by comparing inferred childhood experiences (see Table 1). Individuals classified as continuous-secure showed significantly higher means on all positive inferred parental behaviors and lowest ratings on all inferred negative indices. Few differences on inferred childhood experiences were found between the dismissing, preoccupied, and earned-secure classifications.

**Table 1:** Distribution of Inferred Childhood Experiences across Attachment Groups.

	Dismissing (n = 81)		Preoccupied (n = 16)		Earned-secure (n = 25)		Continuous-secure (n = 86)	
				
	M	SD	M	SD	M	SD	M	SD
Mother								
Loving	3.00^4^	.18	2.58^4^	.36	2.59^4^	.29	5.80^1,2,3^	.15
Rejection	4.56^4^	.23	5.21^4^	.45	5.29^4^	.37	1.80^1,2,3^	.19
Involving-Reversing	1.41^2^	.20	2.85^1^	.39	2.07	.32	1.81	.16
Pressure-to-Achieve	1.79	.20	2.45	.39	1.2	.32	1.38	.16
Neglecting	1.78^2^	.18	3.54^1,3,4^	.35	2.16^2,4^	.29	1.26^2,3^	.14

Father								
Loving	2.81^4^	.18	2.99^4^	.18	2.04^4^	.29	5.51^1,2,3^	.15
Rejection	4.62^3,4^	.23	3.75^3,4^	.46	5.86^1,2,4^	.38	2.09^1,2,3^	.19
Involving-Reversing	1.25^2^	.12	2.28^1,4^	.23	1.62	.19	1.13^2^	.09
Pressure-to-Achieve	1.57	.16	1.87	.31	2.20^4^	.26	1.29^3^	.13
Neglecting	3.45^4^	.28	3.67^4^	.56	3.69^4^	.46	2.06^1,2,3^	.23

Note. Superscripts indicate significantly different means across attachment classifications: 1 = Dismissing, 2 = Preoccupied, 3 = Earned-secure, 4 = Continuous-secure.

The observed differences in inferred childhood experiences emphasize the distinction between earned- and continuous-security. Inferred negative childhood experiences with caretakers may increase the likelihood of problematic substance use despite coherent attachment representations. Given the similarities between individuals classified as dismissing, preoccupied, and earned-secure on the inferred parenting scales, we hypothesized similar rates of problematic substance use across these groups. Based on attachment theory, however, we expected higher rates of treatment participation among those individuals classified as preoccupied or earned-secure and lower rates of participation among individuals classified as dismissing. Individuals classified as continuous-secure were hypothesized to have relatively low rates of substance use problems. We also predicted high rates of treatment participation among individuals classified as continuous-secure due to their hypothesized openness towards emotions and interpersonal relationships [11]. The analyses presented below explicitly tested these assumptions.

### Organized states of mind and substance use problems

The distribution of substance use problems by attachment representations are presented in Table 2. Findings from the logistic regression predicting alcohol abuse/dependence are presented in Table 3. The overall model predicting alcohol abuse/dependence was non-significant, however the contrast comparing individuals classified as continuous-secure versus all other attachment groups approached significance suggesting higher odds of an alcohol diagnosis among individuals classified as dismissing, preoccupied or earned-secure (see Table 3). The overall models predicting illicit substance abuse/dependence were highly significant. The contrast comparing the continuous-secure classification against all other attachment classifications combined was significant for both marijuana and non-marijuana substance abuse/dependence (see Table 3). Comparisons between individuals classified as dismissing, preoccupied, or earned-secure were not significantly different. The odds of receiving a diagnosis of abuse/dependence for illicit substances increased nearly three-fold when classified as dismissing, preoccupied, or earned-secure.

**Table 2:** AAI Attachment Representations and Substance Abuse/Dependence.

	Alcohol Diagnosis^a^ (n = 193)		Marijuana Diagnosis (n = 194)		Non-Marijuana Drug Diagnosis (n = 194)	
			
	No	Yes	No	Yes	No	Yes
Organized Attachment						
Dismissing (Ds)	35 (44%)	44 (56%)	63 (78%)	18 (22%)	66 (82%)	15 (18%)
Preoccupied (E)	7 (44%)	9 (56%)	11 (69%)	5 (31%)	9 (56%)	7 (44%)
Earned-secure (ES)	9 (36%)	16 (64%)	15 (60%)	10 (40%)	20 (80%)	5 (20%)
Continuous-secure (CS)	48 (56%)	38 (44%)	77 (90%)	9 (10%)	78 (91%)	8 (9%)
Unresolved/Disoriented						
Not-unresolved (Not-U)	73 (47%)	81 (53%)	123 (79%)	32 (21%)	130 (84%)	25 (16%)
Unresolved (U)	18 (46%)	21 (54%)	32 (82%)	7 (18%)	31 (80%)	8 (20%)

Note. Ds = dismissing. E = preoccupied. ES = earned-secure. CS = continuous-secure Sample size of 194 for analyses including unresolved/disoriented attachment classification. Percentages are within substance and rows.

^a ^Diagnoses were missing for two subjects due to incomplete data.

**Table 3:** Logistic Regression Predicting Substance Abuse/Dependence from Organized Attachment Contrasts.

	Alcohol Abuse/Dependence^a^			Marijuana Abuse/Dependence^b^			Non-Marijuana Substance Abuse/Dependence^c^		
			
Attachment Contrasts	OR	p	95% CI	OR	p	95% CI	OR	p	95% CI
Ds or E or ES versus CS	1.575	.058	0.984, 2.519	2.89	.001	1.504, 5.587	2.532	.006	1.304, 4.926
E or ES versus Ds	0.930	.787	0.549, 1.576	0.673	.176	0.379, 1.194	0.629	.133	0.344, 1.152
ES versus E	0.878	.693	0.461, 1.672	0.870	.685	0.445, 1.702	1.719	.128	0.856, 3.453

Note. Ds = dismissing, E = preoccupied, ES = earned-secure, CS = continuous-secure.

^a ^Wald χ^2 ^(3) = 4.303, p = .231. ^b ^Wald χ^2 ^(3) = 12.333, p = .006. ^c ^Wald χ^2 ^(3) = 10.586, p = .014.

### Organized states of mind and participation in treatment

The cross-classification table between organized attachment representations and treatment participation are presented in Table 4. Results from the logistic regression models are presented in Table 5. The overall models were significant for both speaking to a professional and outpatient treatment. The attachment contrast comparing individuals classified as dismissing or continuous-secure versus individuals classified as preoccupied or earned secure was significant in both models (see Table 5). The odds of speaking to a professional about mental health problems was 4.5 times more likely if classified as preoccupied or earned-secure. Similarly, participation in an outpatient treatment program increased three-fold when classified as preoccupied or earned-secure. The contrast comparing individuals classified as continuous-secure versus dismissing approached significant for outpatient treatment which was less likely among those classified as continuous-secure (see Table 5). None of the other contrasts were significant.

**Table 4:** AAI Attachment Representations and Participation in Treatment.

	Spoken to a professional		Outpatient treatment	
		
	No	Yes	No	Yes
Organized Classifications^a^				
Dismissing (Ds)	47 (58%)	34 (42%)	49 (61%)	32 (39%)
Preoccupied (E)	4 (25%)	12 (75%)	6 (38%)	10 (62%)
Earned-secure (ES)	5 (20%)	20 (80%)	10 (40%)	15 (60%)
Continuous-secure (CS)	48 (56%)	38 (44%)	64 (74%)	22 (22%)
Unresolved/Disoriented^b^				
Not-unresolved (Not-U)	76 (49%)	79 (51%)	91 (59%)	64 (41%)
Unresolved (U)	19 (49%)	20 (51%)	27 (69%)	12 (31%)

Note. Ds = dismissing, E = preoccupied, ES = earned-secure, CS = continuous-secure. Not-U = not unresolved. U = unresolved.

^a ^n = 208. ^b ^n = 194.

**Table 5:** Logistic Regression Predicting Treatment Participation from Organized Attachment Contrasts.

	Spoken to a professional^a^			Outpatient Treatment^b^		
		
Attachment Contrasts	OR	p	95% CI	OR	p	95% CI
CS or Ds versus E or ES	4.578	.001	2.039, 10.278	3.337	.001	1.618, 6.885
CS versus Ds	1.046	.773	0.770, 1.420	0.726	.056	0.522, 1.740
E versus ES	0.866	.706	0.410, 1.831	1.054	.873	0.553, 2.009

Note. Ds = dismissing, E = preoccupied, ES = earned-secure, CS = continuous-secure.

^a ^Wald χ^2 ^(3) = 17.084, p < .001. ^b ^Wald χ^2 ^(3) = 14.891, p = .002.

### Divergence between need for treatment and participation in treatment

Finally, we examined whether organized attachment representations accounted for lower rates of participation in treatment among individuals having a lifetime substance abuse/dependence diagnosis [43-45]. In the analyses above, we demonstrated that individuals classified as dismissing reported lower rates of treatment participation than individuals classified as preoccupied or earned secure despite equal rates of substance abuse/dependence problems among these three groups. We were interested in examining if this divergence between the presence of substance abuse problems and treatment was statistically significant. Therefore, we identified a sub-sample of individuals having a lifetime diagnosis of alcohol, marijuana, and/or non-marijuana substances (n = 75). We compared treatment participation among individuals classified as dismissing (-1) versus individuals classified as preoccupied or earned-secure (+1). Individuals classified as dismissing were significantly less likely to speak to a professional than individuals classified as preoccupied or earned-secure (see Table 6). The model predicting participation in outpatient treatment showed a marginal effect in the same direction. Therefore, despite the presence of substance abuse/dependence problems, individuals classified as dismissing were less likely to engage in treatment than individuals classified as preoccupied or earned-secure.

**Table 6:** Logistic Regression Predicting Treatment Participation Among Individuals with Substance Abuse/Dependence (n = 75) from Organized Attachment Contrasts.

	Spoken to a professional ^a^			Outpatient Treatment ^b^		
		
Attachment Contrast	OR	p	95% CI	OR	p	95% CI
Ds versus E or ES	2.538	.015	1.200, 5.376	1.779	.090	0.913, 3.472

Note. Ds = dismissing, E = preoccupied, ES = earned-secure.

^a ^Wald χ^2 ^(1) = 6.772, p < .009. ^b ^Wald χ^2 ^(1) = 2.995, p = .084.

## Discussion

Attachment theory provides a useful framework from which appropriate interventions into substance use problems can be developed [1]. Our research questions focused on the association between attachment representations, lifetime prevalence of substance abuse/dependence, and likelihood of participating in treatment. We hypothesized that attachment representations would show predictable associations with problematic substance use and participation in treatment. We further predicted that individuals with certain attachment states of mind would be less likely to seek treatment despite significant problems with substance use.

Our predictions were derived from hypothesized influences of attachment representations on emotional regulation and perceptions about interpersonal relationships [1,3,8,11]. Specifically, individuals with a dismissing state of mind are believed to view relationships as unimportant and minimize distress. Thus, we predicted that dismissing attachment would be associated with a lower likelihood of seeking treatment despite the presence of substance abuse/dependence [39,44,45]. We hypothesized that preoccupied attachment, on the other hand, would be associated with hypervigilance towards interpersonal relationships and distress. Therefore, higher rates of involvement in treatment and substance abuse/dependence were predicted among individuals classified as preoccupied. Finally, we predicted that secure attachment, regardless of inferred early experiences, would be associated with greater openness towards both emotions and interpersonal relationships resulting in a greater likelihood of seeking treatment. We predicted different rates of substance abuse/dependence between the earned- versus continuous-secure classifications [34-37]. Specifically, we anticipated that individuals classified as earned-secure would report higher rates of substance abuse/dependence than those classified as continuous-secure due to greater negative affect associated with the former group.

The findings were consistent with our predictions. Individuals classified as dismissing reported low rates of participation in treatment despite substantial problems with substance abuse/dependence. Individuals classified as preoccupied or earned-secure reported both high rates of substance abuse/dependence and a greater likelihood of seeking treatment. Finally, individuals classified as continuous-secure reported both low levels of treatment participation and substance use problems.

The pattern of findings for dismissing and earned-secure representations identifies attachment representations as an influential factor in the divergence between the presence of problems and likelihood of receiving treatment [39,44,45]. Both groups had similar inferred negative experiences with caretakers during childhood and similar rates of substance use problems; however, individuals classified as earned-secure were more likely to report seeking treatment. One interpretation from our findings is that attachment representations influence an individual's willingness to seek treatment. Individuals classified as earned-secure, despite their negative inferred experiences with caretakers, continue to value interpersonal relationships. Thus, they are more likely to report seeking treatment. An alternative interpretation, although unsubstantiated with these data, is the potential malleability of attachment representations with appropriate treatment [21]. Individuals designated as earned-secure in our sample may have been classified as dismissing or preoccupied prior to entering treatment. The earned-secure classification could thus be a consequence of treatment and not a motivating factor in seeking treatment. Finally, the validity of the earned-secure classification continues to be questioned. It is possible that the actual quality of childhood experiences was not accurately reflected by our inferred experience scales due a negative bias accounting for the low ratings [34-37]. This explanation of earned-security would slightly alter the interpretation of our findings attributing substance use problems to the presence of a negative bias and associated mood disturbance rather than to negative childhood experiences per se. Regardless, our findings add further to the apparent qualitative difference with regard to psychopathology between individuals classified as earned- versus continuous secure.

### Implications for treatment providers

The findings presented in this paper represent merely a snapshot into the complex role of attachment in the therapeutic process [1]. At this point, we are only able to speculate about the nuances introduced by attachment representations and the implication of these nuances for the successful treatment of addiction. For example, our findings suggest that a seemingly uniform outcome (e.g., addiction) may result from qualitatively different external and internal experiences thereby requiring modification of interventions to fit "the developing person" [38]. A continued balance between theory, research, and practice will further advance the development of successful interventions into addiction [1,2,38]. An example of such balance is Flores' [46] incorporation of attachment theory into a model for the treatment of addiction. He conceptualizes addiction treatment as a "time dependent process" [p. 69, [46]] in which attachment-related issues alter the focus and nature of intervention across the different stages of recovery. His depiction of addiction as an "attachment disorder" emphasizes the intricate balance that must be achieved when attempting to shift individuals from substance use towards more adaptive methods of emotional regulation. Although it is not always clinically feasible to fully assess attachment related cognitions, interventionists can be trained to watch for hallmark indicators of behaviors associated specific attachment orientations [1,2]. Such insight by interventionists will increase the likelihood that addicts will be successful in reducing the rewards of substance use (e.g., negative affect reduction) and promote the development of mentalizing abilities (e.g., reflective functioning) that allow greater insight into the motivations behind use [46,47].

### Limitations

This study represents a preliminary examination of the association between attachment and treatment. We used a sample of adoptees which limits generalization of the findings. Although the sample was not selected on psychopathology of the adoptee, the sample was balanced on birth parent antisocial and substance use behaviors which increases risk of these disorders in the adoptees. This biological risk increases the odds of psychopathology among the adoptees limiting generalization to a truly random non-clinical sample.

A second limitation involves incomplete information on the purpose and type of treatment, including referral or self-guided solicitation. The questions used to indicate treatment participation were rudimentary at best. Future studies should delineate what type of professional was seen for treatment (e.g., psychiatrist, psychologist, social worker, counselor), the type of treatment implemented (e.g., pharmacological, cognitive behavioral, group), and duration of treatment. Researchers could then explore if certain treatment programs are better matched to certain attachment representations [1,46].

A third limitation is the inability to pinpoint timing of treatment which is especially relevant when evaluating earned-security. We utilized lifetime estimates for both substance abuse/dependence and treatment. With adequate data, future studies could examine treatment success in terms of recovery from substance abuse/dependence. Exacerbation of substance misuse could also be observed throughout the therapeutic process. It may be possible that use, or desire to use, may increase as working models of attachment are challenged. This would be especially informative in further understanding the complex interplay between attachment representations and substance use problems.

The final limitation pertains to the omission of the unresolved state of mind surrounding abuse or trauma. Unresolved attachment represents a breakdown of the organized attachment system. Although we could predict elevated substance use problems within this group, we could not make specific hypotheses about help-seeking behavior without taking into account secondary organized classifications (e.g., dismissing, preoccupied, earned- or continuous-secure). Unfortunately, the number of individuals classified as unresolved (n = 39) precluded such analyses.

## Conclusion

Our findings demonstrate a potential explanation for discrepancies between substance use problems and treatment of such problems. Referrals for treatment and continued follow-up may become increasingly important depending on one's attachment representations. The process of promoting recovery may also rely heavily on attachment. Assessment of both substance use problems and attachment may improve likelihood of successful recovery from substance use problems.

## Competing interests

The author(s) declare that they have no competing interests.
